# Canine chronic enteropathy—Current state-of-the-art and emerging concepts

**DOI:** 10.3389/fvets.2022.923013

**Published:** 2022-09-21

**Authors:** Albert E. Jergens, Romy M. Heilmann

**Affiliations:** ^1^Department of Veterinary Clinical Sciences, College of Veterinary Medicine, Iowa State University, Ames, IA, United States; ^2^Department for Small Animals, College of Veterinary Medicine, University of Leipzig, Leipzig, SN, Germany

**Keywords:** biomarker, disease modeling, drug discovery, individualized approach, innovation

## Abstract

Over the last decade, chronic inflammatory enteropathies (CIE) in dogs have received great attention in the basic and clinical research arena. The 2010 ACVIM Consensus Statement, including guidelines for the diagnostic criteria for canine and feline CIE, was an important milestone to a more standardized approach to patients suspected of a CIE diagnosis. Great strides have been made since understanding the pathogenesis and classification of CIE in dogs, and novel diagnostic and treatment options have evolved. New concepts in the microbiome-host-interaction, metabolic pathways, crosstalk within the mucosal immune system, and extension to the gut-brain axis have emerged. Novel diagnostics have been developed, the clinical utility of which remains to be critically evaluated in the next coming years. New directions are also expected to lead to a larger spectrum of treatment options tailored to the individual patient. This review offers insights into emerging concepts and future directions proposed for further CIE research in dogs for the next decade to come.

## Update/perspective on disease pathogenesis

**Figure 1:**
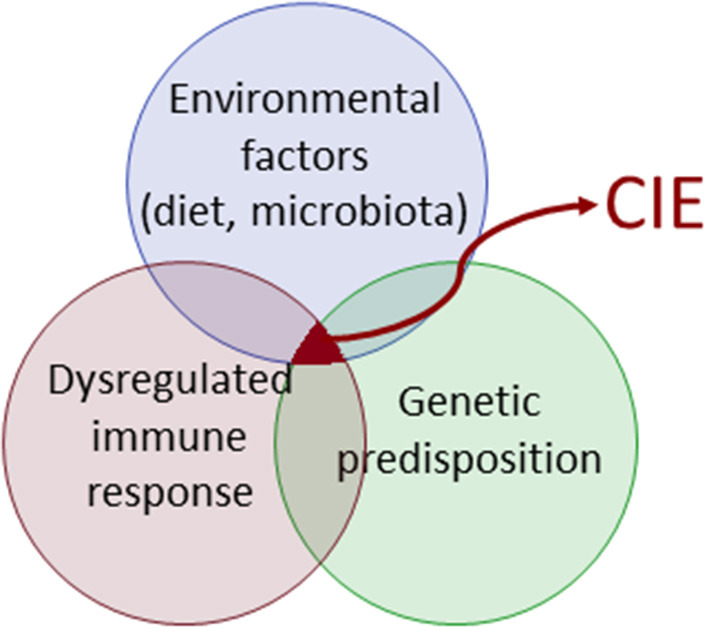
Complex disease pathogenesis in canine chronic inflammatory enteropathy (CIE).

CIE characterizes an exaggerated immune response and has a multifactorial pathophysiology (Figure 1). The immunopathology of CIE results from a complex interplay between elements of innate and adaptive immunity (1). The innate immune system as the first line of host defense is characterized by a system of circulating cells and molecules, sentinel cells, and cellular molecules that orchestrate a complex immune reaction. These signaling pathways including the inflammasome modulate the adaptive immune response, which in turn is subject to adaptive control. Advances in the fields of genomics, microbiome, and metabolomics over the last decade have revealed further important aspects of CIE pathogenesis, but evaluation of the canine CIE exposome (i.e., the life-course of environmental effects or modulators of disease risk) is still in its infancy.

### Disease prevalence and subclassification

Chronic inflammatory enteropathies are diagnosed based on the presence of chronic gastrointestinal signs (≥3 weeks), histopathologic evidence of intestinal mucosal inflammation, and the exclusion of other underlying causes (1–3). The true prevalence of CIE in dogs remains unknown, but it is estimated to present 1–2% of the cases in referral settings (3), and up to 20–30% of veterinary visits in companion animals are reported to be related to vomiting/diarrhea (4). Any dog, regardless of breed, can be affected by CIE (5).

**Figure 2:**
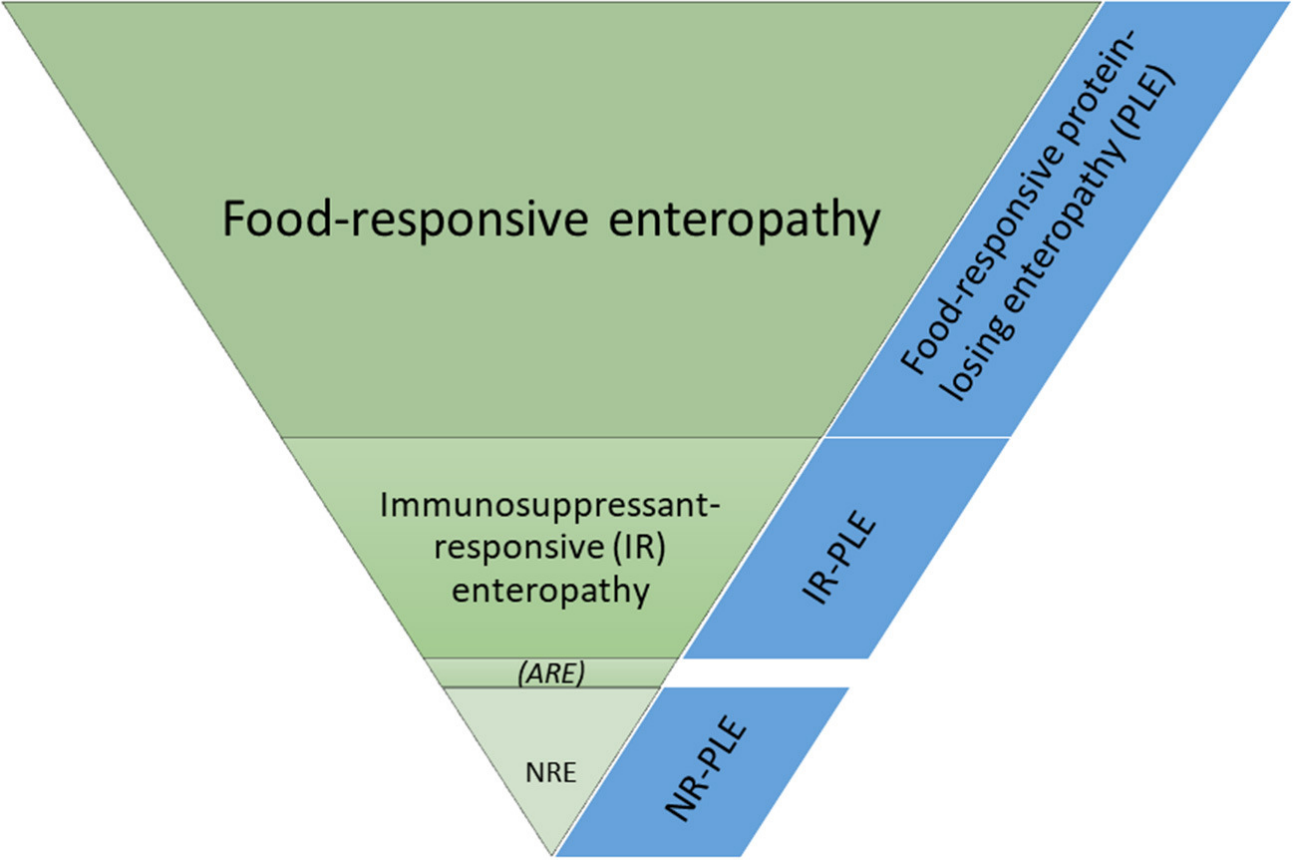
Frequencies of the different subgroups of chronic inflammatory enteropathies in dogs. ARE, antibiotic-responsive enteropathy (idiopathic intestinal dysbiosis); NR, non-responsive; NRE, NR enteropathy.

Canine CIE is currently further subclassified (usually retrospectively) based on the response to treatment. Dogs are categorized as food-responsive enteropathy (FRE) if the clinical signs resolve or significantly improve within 2–4 weeks after starting an elimination diet trial with either a limited-ingredient novel protein and carbohydrate source (commercial or home-cooked diet) or a hydrolyzed protein diet. FRE is the most prevalent group (50–65%) of CIE in dogs (Figure 2), and affected dogs are typically younger and have less severe clinical signs than dogs with other CIE subclasses (2, 3, 6–8).

**Figure 3:**
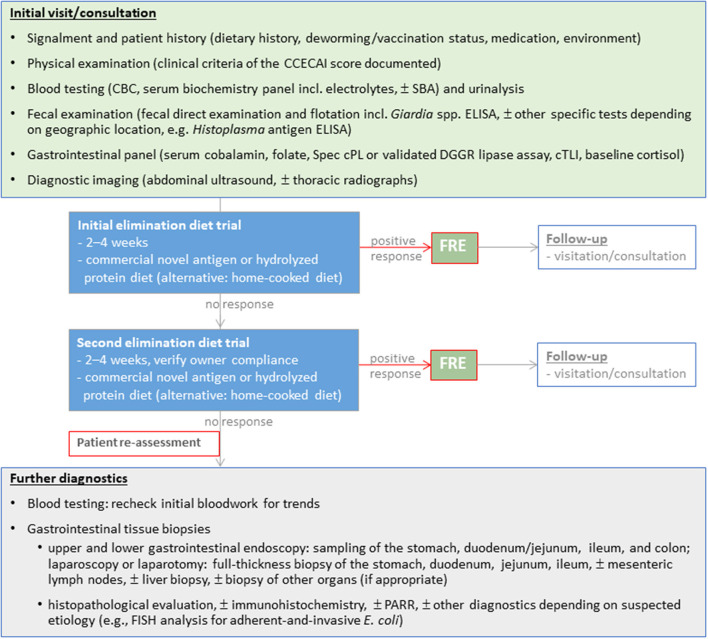
Diagnostic evaluation of dogs suspected with chronic inflammatory enteropathy (CIE). Selection and sequence of the individual diagnostic steps might vary and have to be tailored to the individual patient.

Excellent long-term responses in dogs with FRE can be achieved with commercial hydrolyzed protein or limited-ingredient novel protein diets (2, 9, 10). Use of a hydrolyzed protein diet is the preferred approach by the authors and some clinicians given the clinical impression of a superior response over novel protein/novel carbohydrate diets and the incomplete dietary histories (e.g., treats) in many affected dogs (11). Beneficial effects of feeding a limited ingredient elimination or hydrolyzed protein diet on the gastrointestinal microbiome and metabolome have been demonstrated (12–14). However, the response to different hydrolyzed protein diets varies, and an insufficient clinical response to one diet does not exclude the possibility of remission with another type of hydrolyzed protein diet (15), making a second elimination diet trial essential before either reaching for immunomodulatory drugs and/or more invasive diagnostics (Figure 3).

Dogs that show a marked and long-lasting improvement or resolution of their clinical signs after administering an antibiotic (metronidazole and/or tylosin) for 2 weeks were previously classified as ARE (3). However, antimicrobial treatment can have a long-lasting effect on the intestinal microbiome (16–20), increasing antimicrobial resistance is of major global concern, and most dogs that initially respond to an antibiotic trial will relapse after discontinuing treatment (8). Thus, the true existence of the ARE subgroup (proposed to be renamed “idiopathic intestinal dysbiosis”) of canine CIE and the appropriateness of empirical antimicrobial treatment trials in the diagnostic work-up of canine CIE is currently debated (8, 21, 22).

Dogs that require treatment with glucocorticoids or other immunosuppressive drugs are diagnosed as steroid-responsive (SRE) or immunosuppressant-responsive enteropathy (IRE) (2, 3, 21), which is a diagnosis of exclusion and often referred to as idiopathic inflammatory bowel disease (IBD). Thus, the diagnostic evaluation of dogs suspected to have SRE/IRE is complex and presents a challenge for many owners of affected dogs. Medical treatment of dogs with SRE/IRE usually involves the administration of glucocorticoids (predniso[lo]ne or budesonide) and/or other immunosuppressive drugs (cyclosporine, chlorambucil, or azathioprine) and follows a top-down approach (2, 3, 21, 23). Mycophenolate mofetil is typically not a good choice due to the high rate of gastrointestinal adverse effects (24). Targeted pathway-specific treatment strategies (e.g., therapeutic monoclonal antibodies targeting pro-inflammatory cytokines [TNF-α blockers] and integrins [integrin blockers]) would be desirable glucocorticoid-sparing treatment options for canine SRE/IRE but are currently not available in veterinary medicine and the pathways addressed using biologicals in human gastroenterology are those of Th1-driven intestinal inflammation that has not been shown to be present in dogs.

Approximately 15–43% of dogs classified as SRE/IRE have no adequate response to medical treatment and are categorized as non-responsive enteropathy (NRE), carrying a worse long-term prognosis and a high rate of euthanasia (25, 26). Further studies need to determine if these dogs will benefit from additional or alternative immunomodulatory treatments, including probiotics (e.g., *Enterococcus faecium* or the highly concentrated probiotic strains found in Visbiome), prebiotics (e.g., β-1,3/1,6-D-glucan), or synbiotics (27–34), fecal microbiota transplantation (FMT) (35–37), or stem cell therapy (38).

Protein-losing enteropathy (PLE), as a special form of chronic enteropathies in dogs, can be secondary to a severe diffuse infiltrative intestinal disease process causing lymphatic vascular obstruction (e.g., lymphoplasmacytic enteritis with SRE/IRE) or due to dysfunction of the intestinal lymphatics with primary intestinal lymphangiectasia (39, 40). Based on the response to treatment, PLE is subcategorized as either “food-responsive” PLE (FR-PLE) or non-FR-PLE requiring additional immunosuppressant treatment. Another subtype of PLE in dogs is focal lipogranulomatous lymphangitis (FLL) which is clinically indistinguishable from CIE and PLE, but abdominal ultrasonography typically reveals an increased wall thickness of the distal small intestine (jejunum, ileum, or both) with focal or solitary mass-like serosal lesions that can extend to the mesentery (41, 42).

### Role of genetics—Breed-specific enteropathies

Canine CIE is a multifactorial disease likely caused by complex interactions between mucosal immunity and the environment (i.e., diet, gut microbiota) in genetically susceptible hosts, like human IBD (43, 44). Genetic susceptibility plays an important role in the development of ulcerative colitis and Crohn's disease in humans as genome-wide association studies (GWAS) have identified >100 risk alleles involving genes important in host-microbiome interactions (45, 46). Supporting the notion that host genetics play a similar role in CIE pathogenesis is the observation that certain dog breeds are predisposed to idiopathic IBD (5). However, few causal genetic defects have been identified at this time.

The association between specific breeds [e.g., Boxers and German shepherd dogs (GSD)] and their clinical response to antibiotics points to a potential interaction between host immunity and the microbiota (44). Previous studies have demonstrated upregulated expression of Toll-like receptors (TLR) 2, 4, and 9 in different breeds of dogs with CIE (47, 48), including GSD with IBD where TLR4 was upregulated and TLR5 downregulated at the mRNA level (49). Further studies have identified two non-synonymous single nucleotide polymorphisms (SNPs) in the *TLR4* gene and three non-synonymous SNPs in the *TLR5* gene that are significantly associated with IBD pathogenesis in GSD (50). Interestingly, all the SNPs associated with IBD involved a change in the class of amino acid coded which could change the structural properties of the TLR4 and TLR5 receptors, thereby altering innate immune responses. A preliminary GWAS suggests that five candidate genes associated with IBD in humans could potentially be associated with PLE in Gordon Setters (51). Boxer dogs and French Bulldogs with granulomatous colitis (GC) show sustained remission following eradication of mucosal invasive *E. coli* that are phylogenetically related to an adherent and invasive pathotype seen with Crohn's ileitis (52, 53). While preliminary GWAS in Boxers suggested neutrophil cytosolic factor 2 (NCF2) involved with killing intracellular bacteria as a candidate, subsequent studies now implicate various virulent genes in these bacteria as causing GC-associated mucosal inflammation (54).

Celiac disease in humans causes small intestinal inflammation with high heritability and a strong human leukocyte antigen (HLA) component (55). This strong genetic association reflects the central role of CD4^+^ T cells as the HLA molecules associated with celiac disease bind specific gluten peptides that activate proinflammatory T cells (56). Soft-coated Wheaton terriers (SCWT) have a well-recognized hypersensitivity reaction to different foods that may cause PLE and protein-losing nephropathy (57). Pedigree analysis from 188 SCWT demonstrated a common male ancestor, although the mode of inheritance remains unknown (58). Perinuclear cytoplasmic antibodies (pANCA) are observed with ulcerative colitis in humans (59) and in dogs with IBD, where their occurrence preceded the development of hypoalbuminemia by an average of 2.4 years (60). In a separate study, pANCA was investigated in 65 dogs with CIE at initial examination and in response to treatment. Elevated pANCA levels were observed in 62% and 23% of dogs with FRE and SRE/IRE, respectively; however, there was no difference in pANCA titers between disease groups after treatment (61). Expression of pANCA was detected in 38/104 (37%) dogs with IBD as compared to 4/23 (17%) of dogs with intestinal lymphoma in another investigation (62). These shared observations suggest that autoantibody formation indicative of immune dysgregulation occurs in some dogs with different forms of CIE but is most common in dogs with FRE. Other breed-associated GI diseases having a suspected genetic basis for their occurrence include Shar peis with cobalamin deficiency (63) and Irish setters with gluten-sensitive enteropathy (64).

While the role of genetics in CIE is currently being unraveled, there is evidence in humans and dogs that early life exposures can impact the intestinal microbiome and host response to influence the risk of disease later in life. After birth, the colonizing microbiota promotes development of mucosal immunity (a “weaning reaction”), which imprints the immune system to maintain its responsiveness after weaning and into adulthood. When host-microbe crosstalk is perturbed in early life, inhibition of the weaning reaction leads to pathological imprinting, which causes increased susceptibility to inflammatory pathologies in individuals that have not been exposed to a microbiota, or possibly a dysbiotic microbiota (65, 66). Both experimental (67) and epidemiological studies (68) support this concept of a period of vulnerability for IBD development. One recent study has investigated the long-term effects of parvovirus infection in dogs and its association with chronic gastroenteritis (69). Results of this study suggested that dogs that survived canine parvoviral infection were at increased risk (odds ratio = 5.33) for developing CIE compared to control dogs. Another study found that dogs having an episode of acute hemorrhagic diarrhea were at increased risk (odds ratio = 2.57) for developing chronic GI disease vs. controls later in life (70). These collective observations would support the two-hit model (i.e., the combination of genetic and environmental factors) proposed for IBD pathogenesis (71).

### Immunopathogenesis of chronic intestinal inflammation

The pathogenesis of canine CIE is multifactorial and involves a loss of tolerance to diet and microbial components that cause an aberrant immune response in genetically susceptible hosts (Figure 1). As previously noted, several lines of evidence support innate immune dysregulation as contributing to the development of CIE, like IBD in humans (5, 72). Candidate gene analysis has shown that defects in pattern recognition receptors (*TLR4* and *TLR5*) of the innate immune system are associated with a breed-specific form of CIE in GSDs (50). In a follow-up study, these same investigators showed, in both *in vitro* and *ex vivo* assays, that the canine risk-associated *TLR5* haplotype results in hyper-responsiveness to flagellin vs. the response associated with the CIE risk-protective *TLR5* haplotype (73). Candidate gene mutations and altered mRNA expression were observed for *NOD2* with IBD in GSDs (74, 75).

Regarding adaptive immunity, there has been considerable work to define the role of immune cells and cytokines in CIE. Immunohistochemical investigation of intestinal biopsies has shown no overall difference in total lamina propria (LP) cells in dogs with IBD compared to controls. However, there are differences in the phenotype of LP immune cell subsets with increased numbers of T cells (especially those expressing the αβTCR and CD4^+^), IgG^+^ plasma cells, macrophages, and granulocytes, but fewer mast cells (76). Increased intraepithelial lymphocyte counts are also noted. In dogs with lymphoplasmacytic colitis, there are increased numbers of IgA^+^ and IgG^+^ plasma cells and T cells within the LP and epithelial compartments (77). Analysis of the clonality of B- and T-cell receptors showed reduced diversity in the intestines of dogs with IBD, suggesting expansion of lymphocyte clones with select antigenic specificities (78). Studies of cytokine gene expression in mucosal biopsies have yielded mixed results. One study examining mucosal cytokine expression in GSDs diagnosed with either ARE or IBD and healthy dogs using semi-quantitative reverse transcriptase-polymerase chain reaction (RT-PCR) showed significantly higher mucosal mRNA expression of IL-2, IL-5, IL-12p40, TNF-α, and TGF-β1 in dogs with CIE than in healthy controls (79). A similar study using quantitative RT-PCR (qPCR) showed no differences in IL-2, IL-4, IL-5, IL-6, IL-10, IL-12, IL-18, IFN-γ, TNF-α, TGF-β, and glyceraldehyde-3-phosphate dehydrogenase expression in dogs with and without CIE (80). Separate reviews on this topic have concluded that most dogs with CIE express a balanced Th1/Th2 cytokine profile (81, 82). There is also no evidence of a Th17 signature in dogs with CIE (83). This observation in canine CIE contrasts to human IBD, where distinct Th1 (i.e., cell-mediated) and Th2 (i.e., humoral) cytokine polarization occurs in Crohn's disease and ulcerative colitis patients, respectively (84).

Evidence of immunological dysfunction in CIE also includes the expression of mucosal nitrite, a precursor of the inflammatory mediator of nitric oxide (NO) in dogs with idiopathic IBD (85) and increased numbers of inducible nitric oxide synthase (iNOS)-expressing cells associated with PAS^+^ macrophages in biopsies of dogs with GC (86). Other systemic and locally produced biomarkers of inflammation, including C-reactive protein (CRP), pANCA, and fecal calprotectin, are discussed in a comprehensive review (87). A study using gene expression microarray to investigate intestinal gene expression profiles in dogs with CIE compared to healthy controls showed >1,500 genes (85%) to be downregulated with CIE, including neurotensin, fatty acid-binding protein 6, fatty acid synthase, aldehyde dehydrogenase-1 family member B1, metallothionein, and claudin 8 (88). Few genes were upregulated in dogs with CIE, including those involved in extracellular matrix degradation (matrix metallopeptidases 1, 3, and 13), inflammation (TNF, IL-8, peroxisome proliferator-activated receptor-γ, and S100 calcium-binding protein G), iron transport (solute carrier family 40 member 1), and immunity (CD96 and carcinoembryonic antigen-related cell adhesion molecule [CEACAM]) (88). Dogs with severe CIE and PLE had the largest number of differentially expressed genes. Results of qPCR testing for select genes confirmed the microchip analysis.

MicroRNAs (miRNAs) are small, non-encoding RNAs that regulate gene and protein expression. As such, they play a crucial role in the development of cells involved in innate and adaptive immunity, and in regulating the immune response (89). Studies in human IBD indicate that tissue miRNA profiles are dysregulated in patients with active ulcerative colitis, Crohn's ileitis, and Crohn's colitis (90–92). Increased expression of miR-21 is the most consistently replicated finding in human IBD (93). Tissue and serum miRNA levels of dogs with CIE have been recently investigated. Compared to healthy controls, dogs with large intestinal IBD showed increased miR-16, miR-21, miR-122, and miR-147 expression in the colonic mucosa and serum, while the expression of miR-185, miR-192, and miR-223 was decreased (94). Furthermore, the expression of serum miR-192 and miR-223 correlated to both clinical disease and endoscopic score. These observations suggest that miRNAs are involved in the pathogenesis of canine idiopathic IBD, like human IBD.

A key element in the pathogenesis of CIE is an impaired intestinal epithelial barrier contributing to the loss of immunological tolerance. Different studies using selective sugar permeability testing to evaluate intestinal permeability in dogs with CIE have yielded conflicting results (95–97). More accurate estimates of intestinal permeability have been made using iohexol, a radiographic contrast medium, administered orally and measured in the serum of healthy dogs (98). Future studies investigating the value of iohexol clearance as an intestinal permeability test in dogs with CIE are warranted. Techniques to evaluate intestinal barrier function *in vivo* in humans include orally ingested probes (polyethylene glycols, ^51^Cr-EDTA, sugars) assessed in urine and measurement of fatty acid bindings proteins, glucagon-like peptide (GLP)-2, and LPS-binding protein (LBP) concentrations in serum (99). Apart from sugar permeability testing, these other function tests have not been critically assessed in dogs with CIE.

### Hypovitaminosis B_12_

Assessment of the cobalamin (vitamin B_12_) status as a marker of intestinal absorptive capacity is of diagnostic and therapeutic value in small animal medicine (100). Cobalamin is a water-soluble B vitamin available through mostly dietary sources of animal origin and absorbed mostly *via* specific receptors that internalize the intrinsic factor (IF)-cobalamin complex in the distal small intestine (100). The intestinal microbiota can provide small amounts of cobalamin, but the body hardly benefits from this source because of the distal production site. Body stores, particularly in the liver, can ensure cobalamin availability for several months (100).

Cobalamin deficiency in dogs is currently determined by routine serum cobalamin measurement, often together with serum folate (vitamin B_9_), for which several commercial tests are available. An automated chemiluminescence test is routinely used in Europe and North America, and the reference intervals for serum cobalamin concentrations in dogs used by the various veterinary diagnostic laboratories are comparable (100).

Methylmalonic acid (MMA) is a metabolite that accumulates when the activity of cytosolic methylmalonyl-CoA mutase is decreased because of intracellular cobalamin deficiency (100). Thus, paired methylmalonic acidemia and/or aciduria due to an intracellular lack of cobalamin is a superior test for cobalamin deficiency at the cellular level (101, 102), and ideally, serum cobalamin and serum or urine MMA are used to properly assess the cobalamin status in dogs. However, measurement of MMA is not routinely performed in small animal medicine, but serum MMA tests have recently become available through some veterinary laboratories. Careful interpretation of serum MMA concentrations is warranted in dogs with renal insufficiency (103). Evaluation of serum holo-transcobalamin (cobalamin-saturated transcobalamin) in addition to or instead of serum cobalamin and MMA concentrations may more accurately reflect the cobalamin status (104). Suboptimal serum cobalamin levels were associated with higher serum transcobalamin concentrations than normocobalaminemia in dogs with CIE, but further evaluation of the clinical utility of this marker in dogs is needed.

Hypocobalaminemia or cobalamin deficiency can have several causes, the most common in dogs being chronic gastrointestinal diseases and exocrine pancreatic insufficiency [because in dogs, IF is primarily produced by the pancreas (105)]. Commercial dog foods are fortified with cobalamin; thus, dietary causes of cobalamin deficiency are unlikely unless fed a vegetarian or vegan diet without added cobalamin. Hypocobalaminemia frequently occurs in dogs with CIE (19–54%) and affects 22–75% of dogs with PLE (7, 25, 103, 106–108), which has been presumed to result from distal small intestinal cobalamin malabsorption due to ileal cobalamin receptor deficiency, secondary small intestinal dysbiosis (with increased utilization of cobalamin by the intestinal microbiota), or both. Contrary to the previously proposed pathogenetic mechanism, a recent study showed that cobalamin receptor downregulation is not the primary cause of hypocobalaminemia in canine CIE. Overexpression of the cobalamin receptor subunits amnionless (AMN) and cubilin (CUBN) in the ileum was detected and presumed to be a mechanism to compensate for CIE-associated hypocobalaminemia (109). Hypocobalaminemia was also shown to be a negative prognostic factor in dogs with CIE and to be associated with hypoalbuminemia (7, 25). However, documenting normoalbuminemia does not rule out a diagnosis of CIE. Concurrent hyperfolatemia and hypocobalaminemia suggest intestinal dysbiosis (110).

Subnormal or low normal serum cobalamin concentrations (i.e., < 350–400 ng/L) are often associated with increased serum MMA concentrations (101). These indicate a need for cobalamin replacement therapy to replete body cobalamin stores and aid with mucosal regeneration. Supplementation of cobalamin is recommended with cyanocobalamin. It was previously recommended to be given parenterally [50 μg/kg SQ once weekly for 6 weeks and another dose 1 month later, then continued every 2–4 weeks if needed (111)] while also addressing the underlying CIE. However, recent studies suggest that supplementation with oral cyanocobalamin (50 μg/kg PO q24 h for at least 12 weeks) can be as effective as parenteral administration and present a simple, non-invasive, and pain-free alternative to weekly SQ injections (112–114). Measurement of serum cobalamin concentration is recommended 1 month after the last dose to decide whether to continue cobalamin supplementation every 2–4 weeks (111). Successful treatment leads to a normalization of the biochemical findings and in most dogs, supranormal serum cobalamin concentrations. If supplementation with cyanocobalamin is not effective and serum cobalamin concentrations remain below the target range, hydroxycobalamin (presenting the natural form of cobalamin) given at a dose of 1,000 μg (for a medium-sized dog) IM q14d could be considered an alternative (115), followed by a recheck of serum cobalamin to screen for successful normalization. Oral cobalamin supplementation may particularly be effective in dogs with CIE and very high expression of the ileal cobalamin-IF-receptor, but passive diffusion across the intestinal mucosal epithelium could also explain why oral cobalamin supplementation might be effective.

### Microbiome and metabolome

Intestinal dysbiosis is associated with mucosal inflammation and GI dysfunction in dogs with CIE. Microbial imbalances are characterized by broad shifts in the composition of microbes, reduced species diversity, and changes in the relative proportion of select microbial members compared to feces from healthy dogs (116–118). Most sequencing-based studies indicate reduced relative abundances of Bacteroidetes, *Fusobacterium* spp., and Firmicutes, and an increased abundance of Proteobacteria in fecal samples of dogs with CIE (118). A study using fluorescence *in situ* hybridization (FISH) to characterize the mucosal biogeography in colonic biopsies of dogs with CIE showed affected dogs to have decreased numbers of total bacteria within colonic crypts but no difference in total bacterial counts on the mucosal surface as compared to healthy control dogs (119). Numbers of mucosal *E. coli* were increased in dogs with CIE, like in a previous report (120). In contrast, the numbers of mucosal *Helicobacter* spp and *Akkermansia* spp were decreased in both colonic crypts and the mucosal surface, suggesting that those bacteria are beneficial resident species in the canine colon. GC in Boxers and French bulldogs is a unique form of CIE characterized by adherent and invasive *E. coli* (AIEC) residing within the colonic mucosa. A causal role for these bacteria in GC pathogenesis is supported by the association of remission with eradicating invasive *E. coli* when enrofloxacin was used (53, 121). Other studies in GSD with CIE have shown a unique dysbiosis characterized by increased *Lactobacillus* spp. relative to Greyhound controls (49) and a complex but variable dysbiosis in dogs with tylosin-responsive diarrhea (ARE) (122). Whether intestinal dysbiosis is a cause or consequence of mucosal inflammation in dogs with CIE remains unknown.

Assessment of the gut microbiome is best performed using molecular methods, including sequencing techniques detecting 16S ribosomal RNA genes and metagenomic shotgun sequencing (123). As both next-generation and metagenomic sequencing methods are expensive and time-consuming to perform, more rapid results with lower costs may be realized using a qPCR assay-based algorithm called the fecal dysbiosis index (DI) (124). The DI measures the abundance of 7 bacterial taxa (*Fecalibacterium* spp, *Turicibacter* spp, *E. coli, Streptococcus* spp, *Blautia* spp, *Fusobacterium* spp, and *Clostridium hiranonis* [now *Peptacetobacter hominis*]) and the total bacterial abundance in canine feces. The DI calculates a single number that expresses the extent of intestinal dysbiosis present: a DI < 0 indicates a normal microbiota while a DI >2 indicates dysbiosis (124). The DI can be used to assess normal vs. abnormal microbiota at a single time point or longitudinally in response to disease progression or therapeutic intervention (125–127).

Along with changes in microbial composition, functional changes in the microbiome have also been reported with canine CIE. In one study, dogs with idiopathic IBD exhibited differences in their fecal microbiome and serum metabolome compared to healthy controls (128). The serum metabolites 3-hydroxybutyrate, hexuronic acid, ribose, and gluconic acid lactone were significantly more abundant in dogs with IBD suggesting the presence of oxidative stress. Moreover, while the IBD dogs improved clinically with treatment, this was not accompanied by improvement in the microbiome or serum metabolite profiles. One group of metabolites of particular interest to host health is the short-chain fatty acids (SCFAs; e.g., butyrate, acetate, and propionate) derived from bacterial fermentation of complex carbohydrates that modulate mucosal immune responses. SCFAs are an essential energy source for colonocytes; they enhance epithelial barrier function by strengthening tight junctions and regulate the size and function of the regulatory T-lymphocyte pool to protect against mucosal inflammation (129–131). Relative abundances of SCFA-producing bacteria are decreased in dogs with both acute and chronic diarrhea (132). Examples of other beneficial microbe-derived metabolites include B vitamins and vitamin K.

Select amino acids, including tryptophan, are reduced in canine CIE (133). Tryptophan is an essential amino acid in dogs and serves as a precursor for kynurenine, serotonin, melatonin, and indole. Increased tryptophan catabolism limits the production and availability of serotonin which is an essential neurotransmitter mediating GI secretion, motility, and pain. Tryptophan also serves as a precursor to producing indole compounds by the gut microbiota that enhance mucosal homeostasis by improving gut permeability and increasing mucin production (134). Depletion of tryptophan occurs in dogs with PLE, whereas dogs with idiopathic IBD have decreased indole compounds present in their feces (133).

Intestinal dysbiosis can also trigger chronic mucosal inflammation and bile acid (BA) dysmetabolism (125). Luminal BAs are essential for lipid digestion and play major roles in maintaining mucosal homeostasis through their antimicrobial actions. In the healthy gut, select bacteria are required for deconjugation and dehydroxylation of primary BAs into secondary BAs. With dysbiosis, the decreased abundance of *Peptacetobacter* (*Clostridium*) *hiranonis*, a bacterium associated with normal BA metabolism, causes increased concentrations of primary BAs in the colon, which are pro-inflammatory and may cause secretory diarrhea (134). Bile acid diarrhea (BAD) occurs in a subset of humans with Crohn's disease and diarrhea-predominant irritable bowel syndrome (135). A recent report describes treatment of presumptive BAD in two dogs with CIE using the bile acid sequestrant cholestyramine (136). Taken together, these observations suggest that intestinal dysbiosis perturbs BA metabolism which in turn further promotes intestinal inflammation and GI signs. Dogs with CIE that are non-responsive to traditional therapies might respond to empirical treatment with bile acid sequestrants. Lastly, clinical remission accompanied by improved microbial composition, expansion of BA-producing bacteria, and increased levels of secondary BAs has been reported in dogs with FRE fed a hydrolyzed protein diet (14).

## Update/perspective on diagnostic strategies

The diagnostic work-up of dogs with suspected CIE remains complex (Figure 3) and comprises a thorough patient history, physical examination, clinicopathologic evaluation and diagnostic imaging to rule out other gastrointestinal or extra-gastrointestinal etiologies that can mimic CIE (1, 3, 21). Histologic evidence of mucosal inflammation confirms the diagnosis, and appropriately designed treatment trials aid in classifying the disease to determine the optimal individual treatment plan (3, 21). Disease-independent clinical scoring systems can semi-objectively assess the clinical disease severity at the time of diagnosis and monitor patient improvement during treatment (25, 137). Several biomarkers can aid in the diagnostic evaluation and/or monitoring dogs with CIE (87).

### Gastrointestinal endoscopy, mucosal assessment, and mucosal healing

The decision to perform GI endoscopy in dogs with CIE is best determined on a case-by-case basis. Treatment trials are the best tools to differentiate the varied forms of CIE as clinical history, laboratory findings, biomarkers of inflammation, and histologic abnormalities have not proven reliable to distinguish one form of CIE from another. While many dogs will quickly respond to elimination dietary trials, non-responders will require reassessment and further diagnostic testing including GI endoscopy to rule out other causes for chronic GI signs (Figure 3). Endoscopic biopsy of the GI tract provides rapid, relatively non-invasive, direct assessment of mucosal lesions and permits directed biopsy for histopathologic evaluation (138, 139). Other advantages of flexible endoscopy include the collection of multiple tissue samples from different alimentary (stomach, duodenum/proximal jejunum, ileum, and colon) sites and minimal risks (perforation) associated with the procedure. It is particularly useful in diagnosing infiltrative, erosive, or anatomic (lymphangiectasia) lesions seen with different forms of CIE, but especially SRE/IRE or IBD, as well as low-grade intestinal lymphoma (140). Severe weight loss, prolonged anorexia, poor body condition, hypoalbuminemia, ultrasonographic evidence of substantial infiltrative disease, or a combination of these findings indicates that GI endoscopy should be performed earlier during diagnostic testing rather than after (unsuccessful) therapeutic trials. Practical considerations for endoscopy include the role of ileal biopsy (141), acquisition of good quality tissue specimens (142), proper tissue processing that avoids artifacts and permits optimal tissue orientation in the laboratory (143), and the effect of endoscopic forceps on quality of mucosal biopsy (144).

Careful and thorough examination of mucosal surfaces should be performed and documented using standardized reporting. In this regard, the original World Small Animal Veterinary Association (WSAVA) guidelines recommend using a standardized endoscopic report form with check boxes to identify the presence/severity of mucosal lesions when endoscopy is performed (https://wsava.org/global-guidelines/gastrointestinal-guidelines/) (145). Determining the presence of mucosal inflammation at diagnosis or following treatment is an important consideration in humans with IBD. Measures of mucosal erythema, increased granularity, vascular pattern (colon), spontaneous bleeding, and/or mucosal damage (mucus, fibrin, exudates, erosions/ulceration) have been used in clinical trials (146, 147). Mucosal healing (MH) is an important treatment endpoint in human IBD and achieving MH has been demonstrated to improve patient-related outcomes (146, 148). Broadly, MH represents the absence of friability, erosions, and ulceration in patients with ulcerative colitis and Crohn's disease.

There are few studies evaluating the GI mucosal appearance of dogs with CIE following treatment. One prospective single-center study evaluated the sequential treatment response of 70 dogs with CIE placing them into FRE, ST (steroid-treated), and PLE cohorts (25). Gastroduodenoscopy and colonoscopy were performed in all dogs at diagnosis (except those with panhypoproteinemia), while repeat endoscopy was performed in ST dogs and a subset of ST dogs requiring treatment escalation. Endoscopic indices included the presence of mucosal erythema, friability, granularity, ulceration, white speckling, and degree of insufflation. While there was no difference in endoscopy scores among the FRE, ST, and treatment escalation groups, an increased endoscopy score indicating severe duodenal inflammation was associated with negative long-term outcome. A similar study evaluated the clinical, endoscopic, and histopathologic response to treatment in non-hypoproteinemic dogs with lymphoplasmacytic enteritis (149). Different macroscopic lesions to the gastric and duodenal mucosa were identified at diagnosis and following 120 days of diet and medical (prednisone and metronidazole) therapy. Seventy-five percent of dogs showed improvement in endoscopic gastric and duodenal lesions (defined as a reduction in the total macroscopic score post-treatment) indicative of MH. These two studies demonstrate the feasibility of using GI endoscopy to assess MH and outcome in dogs with CIE.

**Figure 4:**
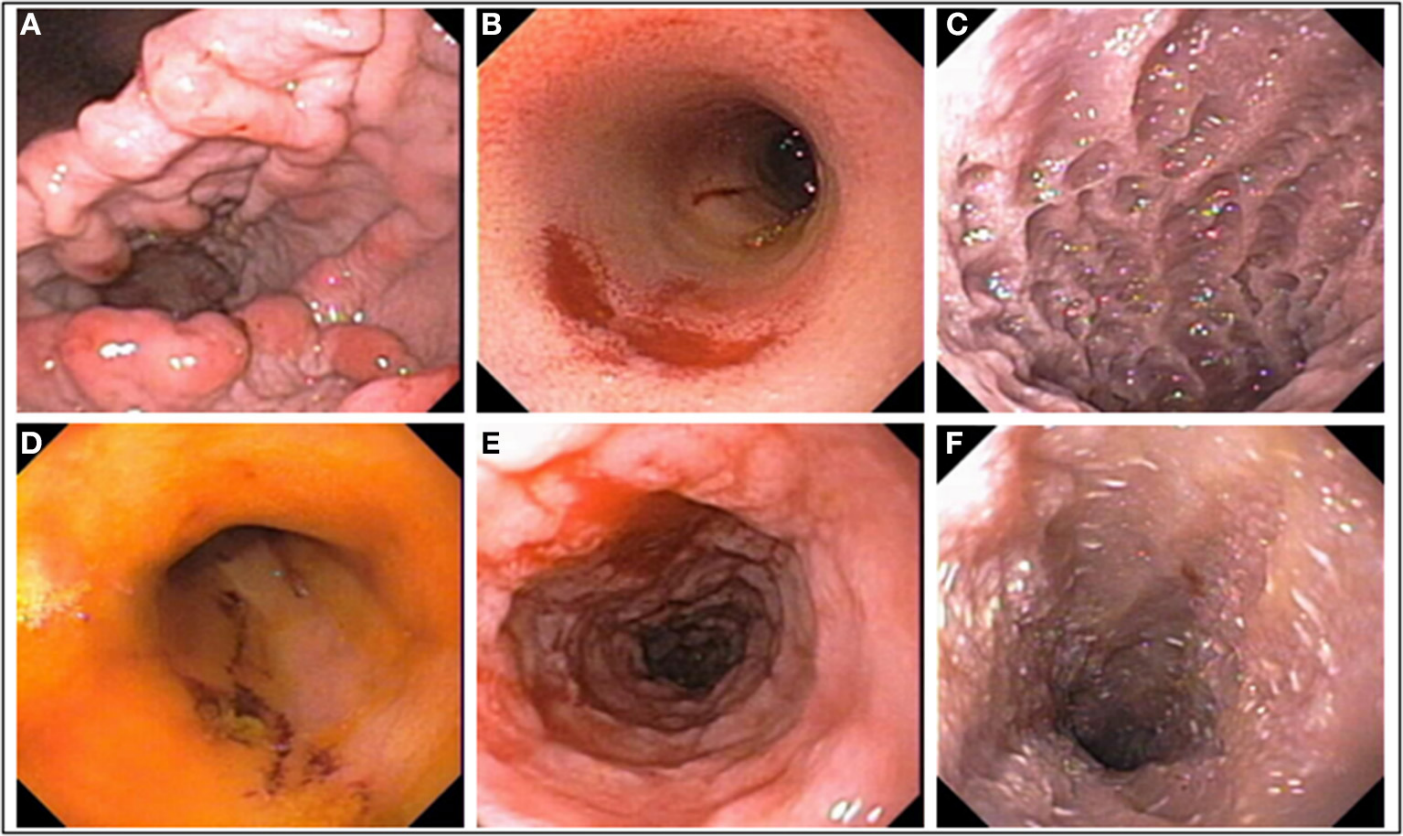
Mucosal abnormalities used in calculation of the simple endoscopic score. **(A)** Gastric granularity, **(B)** duodenal erosions, **(C)** duodenal granularity, **(D)** ileal erosions, **(E)** colonic granularity, and **(F)** lymphangiectasia, duodenum.

The WSAVA endoscopic examination reporting forms for upper and lower GI endoscopy include various descriptors (hyperemia, edema, discoloration) and subjective gradations (mild, moderate, severe) that may make it difficult to accurately quantitate mucosal inflammation. Furthermore, studies evaluating the interobserver agreement for grading mucosal lesions amongst endoscopists using the WSAVA endoscopic score are lacking. One recent study investigated the interobserver agreement in the endoscopic assessment of mucosal appearance of the GI tract to design and validate a simple and easy-to-use endoscopic scoring system for dogs with idiopathic IBD (150). Validation studies using endoscopic videoclips to assess the same endoscopic parameters defined by quantitative (lesion number and severity) vs. qualitative (present or absent) methods (Figures 4A–F) showed moderate-to-substantial agreement between experienced endoscopists. Since the quantitative and qualitative scores of mucosal appearances were very similar, an endoscopic activity score that incorporates only the presence/absence of four mucosal variables, and not their magnitude or the extent of their severity, is proposed for use in the endoscopic assessment of mucosal inflammation (Table 1). Future studies are required to confirm correlation of the simple endoscopic score to clinical activity and laboratory biomarkers of inflammation in dogs with CIE.

**Table 1:** Simple endoscopic score for canine chronic enteropathy [modified from (151)].

**Lesion**	**Score^b^**	**Description**
Friability	0	Absent
	1	Present
Granularity	0	Absent
Erosions	1	Present
	0	Absent
White speckles/spots^a^	1	Present
	0	Absent
	1	Present

^a^Indicative of lymphatic dilatation.

^b^Maximum gastroscopy score: 3 (assessing only criteria 1–3); maximum enteroscopy score: 4; maximum colonoscopy score: 3 (assessing only criteria 1–3).

### Histologic guidelines for gastrointestinal inflammation

Diagnosis of intestinal inflammation in dogs with CIE has historically posed great challenges for clinicians and veterinary pathologists. While intestinal biopsies can be acquired endoscopically or surgically, GI endoscopy is most often utilized. Several fundamental issues in the diagnostic process have included: (1) How can small and fragile endoscopic specimens of variable quantity from different regions of the GI tract be used for histologic assessment? (2) What constitutes an acceptable biopsy with regards to size and diagnostic quality? (3) Are standardized grading schemes of value in defining histologic inflammation? If so, what are the key criteria used for assessment (i.e., type/quantity of cellular infiltrate, mucosal architectural changes, other changes) and current recommendations for their use? (4) Is there a relationship between histologic changes and GI signs? and (5) Is there a relationship between histologic changes and response to treatment? To address these concerns, numerous histologic grading schemes characterizing the nature and severity of GI inflammation have been designed. Within most of these model systems, there is an emphasis on the type and severity of lamina propria cellularity that is variable and subjectively graded as normal, mild, moderate, or severe. It is now recognized that different types of cellular populations (e.g., lymphoplasmacytic, eosinophilic, and granulomatous) may overlap and occur in different types of GI diseases. For example, lymphoplasmacytic enteritis is a common feature in dogs with idiopathic IBD (25, 77, 151, 152) and is described in association with PLE (153). The reliance on mucosal cellularity as a key feature of GI inflammation has meant that abnormalities in mucosal architecture (i.e., epithelial lesions, villous morphology, lymphatic dilatation, fibrosis, and crypt changes) have been overlooked but may correlate best with inflammatory markers and clinical severity of GI disease (53, 154). The quality of endoscopic specimens (influenced by operator experience in detecting mucosal lesions) (143) and the effect of tissue processing (155) are additional limitations affecting histologic assessment. These findings and the observation that substantial variation occurs between pathologists when interpreting GI histologic findings (156) emphasized the need to develop uniform guidelines for evaluating GI histologic findings. Against this background, the WSAVA GI Standardization Group provided the first comprehensive guidelines for the standardized interpretation of inflammatory changes in the GI mucosa of dogs and cats (145). These guidelines included a visual and textual description of morphologic and inflammatory changes in the stomach, duodenum, and colon ranging from mild to severe as compared to normal GI histology.

A limitation of the WSAVA score is that it did not consider goblet cells as a morphologic parameter, where their numbers decrease with inflammation but increase in response to successful treatment (121, 154). Using the 253 animals from the original WSAVA study, a simplified histopathologic model for GI inflammation was designed to improve the consistency of interpreting GI inflammation between pathologists (157). This refined and reductionist model used statistical analysis to identify those WSAVA parameters that showed the least interpretive variation between pathologists of the original report (Table 2). The simplified model incorporates the number of goblet cells as a morphologic feature to grade when evaluating colonic endoscopic specimens. While histopathologic guidelines for interpretation of ileal mucosal specimens were not proposed in the WSAVA scheme, it is recommended to use the simplified model criteria of duodenal inflammation for microscopic evaluation of ileal biopsy specimens. Besides the original report, this model has been applied successfully to characterize the severity of histologic inflammation in biopsy-diagnosed CIE with and without hypoalbuminemia (158) and has shown to positively correlate with clinical disease activity in dogs with idiopathic IBD (159).

**Table 2:** Histopathologic features of the simplified histopathologic model for gastrointestinal inflammation [from (157)].

**Organ**	**Histologic feature**
Stomach	Intraepithelial lymphocytes (IEL)
	Lamina propria (LP) infiltrate
	Fibrosis
	Nesting
	Mucosal atrophy
Small intestine	Villous atrophy/stunting
	Epithelial injury
	Crypt dilation/distortion
	IEL
	LP infiltrates
Colon	Epithelial injury
	Crypt dilation
	Fibrosis/atrophy
	LP infiltrates
	Goblet cell number

Endoscopic biopsy is often performed in dogs with chronic or intermittent GI signs that fail to respond to sequential treatment interventions. In these instances, it is expected that the histologic findings contained in tissue samples will confirm a diagnosis and guide treatment decisions in these patients. However, separate trials performed by different investigators have failed to show a convincing association of histopathologic findings in endoscopic specimens with clinical disease activity, biomarkers of inflammation, or response to therapy and outcome in dogs with idiopathic IBD (25, 26, 32, 150, 160, 161). The failure to demonstrate an association in these earlier studies might relate to the use of non-standardized histologic grading systems and/or differences in study design. In one recent study, the simplified GI scoring system demonstrated significant correlations with histologic scores and clinical indices (25). Compared to earlier grading schemes, the simplified score showed improved utility in correlating histologic features (total summative histologic scores in the duodenum and colon and select histologic scores) to IBD clinical activity.

Other factors contributing to potential discordance between histologic findings and clinical signs include the quality of endoscopic specimens and failure to biopsy the intestinal compartment most affected by a pathologic process. The effect of sample quality in detecting select histologic lesions in gastric and duodenal biopsies of dogs was investigated in one study showing that endoscopic biopsy quality (i.e., adequate vs. inadequate specimens) significantly affects the sensitivity to detect certain histologic lesions (142). As the quality of the endoscopic sample improved from inadequate to marginal to adequate, fewer endoscopic samples were required to diagnose duodenal lesions and most gastric lesions. Other investigations have focused on the value (extent of agreement in histologic changes) of simultaneous duodenal and ileal biopsies in dogs with CIE. In a separate study, there was poor agreement between pathologic lesions from duodenal vs. ileal biopsies, with a greater number of histologic abnormalities detected in the ileum of dogs with concurrent small and large intestinal diarrhea (162). Finally, in dogs with chronic small intestinal enteropathy, there was only slight agreement between duodenal and ileal histologic scores, except for the correlation of hypoalbuminemia with ileal lacteal dilatation and the number of ileal intraepithelial lymphocytes (141). These combined observations indicate that collection of concurrent duodenal and ileal biopsies is warranted in dogs with CIE requiring GI endoscopic biopsy.

Changes in the ultrastructural lesions of duodenal biopsies (i.e., enterocytes) have been reported in dogs with diet-responsive CIE before and after dietary treatment (163). Using both qualitative and quantitative measures of ultrastructural features, dietary therapy in dogs with FRE decreased the mean mitochondrial injury score and intermicrovillar space of intestinal epithelia and increased the mean microvillar height indicating recovery of enterocyte mitochondrial and brush border health.

### Biomarkers of inflammation

Clinically useful biomarkers help detect disease, determine the severity of the disease process, and monitor the response to treatment. Several inflammatory biomarkers have been researched during the last decade and hold significant promise as diagnostic and/or monitoring tools in canine CIE (87). These include CRP, calprotectin, S100A12, N-methylhistamine (NMH), 3-bromotyrosine (3-BrY), and soluble receptor of advanced glycation end-products (sRAGE).

*Serum C-reactive protein (CRP)* concentration is a non-specific marker of inflammation (87). CRP is a positive type II acute-phase protein expressed in the liver in response to infection, inflammation, or cancer (164, 165). Several assay formats are available for the quantification of canine CRP in serum (166–172), which have reference intervals of approximately < 10 mg/L (173). A high-sensitivity CRP (hs-CRP) assay with improved sensitivity for lower serum CRP concentrations would be highly desirable but is currently not routinely available in small animal medicine (174).

Due to the high inter-individual biological variability of serum CRP concentrations in dogs (175), its utility as a diagnostic biomarker in canine CIE is limited. Still, a serum CRP concentration ≥9.1 mg/L was highly specific (100%) to detect dogs requiring anti-inflammatory or immunosuppressive treatment (SRE/IRE and NRE) among all CIE cases including those responding to an elimination diet (FRE) or antimicrobial treatment (ARE) (87, 108). Serum CRP is also useful as a surrogate biomarker to assess disease progression and response to treatment in canine CIE (108, 176, 177), but serum CRP concentration needs to increase or decrease at least 2.7-fold to be considered a clinically relevant change (175). Increased serum CRP levels are not specific for the gastrointestinal tract, but the marker can be used in corticosteroid-treated dogs.

*Fecal calprotectin* concentration appears to be a useful biomarker of intestinal inflammation in dogs (108, 177, 178). The calprotectin protein (S100A8/A9) complex belongs to the damage-associated molecular pattern (DAMP) molecules (alarmins) and accumulates at sites of inflammation (179). It is a ligand for Toll-like receptor (TLR)-4 (180), which plays a role in the pathogenesis of canine CIE (44, 46). The stability of the protein complex allows for its extraction and measurement in canine fecal samples (181, 182).

Using a cut-off concentration of ~50 μg/g, fecal calprotectin is a good surrogate marker of severe clinical disease in canine CIE (108, 177, 178). Fecal calprotectin may help predict the response to treatment in dogs with CIE, with levels ≥15.2 μg/g distinguishing partial responders or non-responders (NRE) from SRE/IRE dogs that achieve complete clinical remission with high sensitivity (80%) and moderate specificity (75%) (108). Careful selection of canine patients for fecal calprotectin testing is important to gain relevant information from this biomarker as increases also occur with acute gastrointestinal inflammatory conditions (183). Fecal calprotectin measurement in dogs is currently not routinely available, but a human immunoturbidimetric assay that can be run on standard laboratory chemistry analyzers has shown promise for fecal samples from dogs (182, 184).

*Fecal S100A12* (calgranulin C) is another sensitive and specific marker of localized inflammatory processes in dogs (107, 185) and has a cellular distribution similar to calprotectin (179). The S100A12 protein is a DAMP molecule with several target proteins, including the receptor for advanced glycation end-products (RAGE) with a central role in innate and acquired immune responses (186). S100A12 is stable in fecal samples and can be measured after extraction (187).

The severity of clinical signs and endoscopic lesions, but not histopathologic changes, correlates with fecal S100A12 levels in dogs with CIE (107, 185), and the marker may also aid in predicting the response to treatment and disease outcome. Dogs with CIE and a fecal S100A12 concentration < 490 ng/g are very likely to respond to an elimination diet (and/or antibiotic) trial (i.e., diagnosis of FRE/ARE) and not require anti-inflammatory or immunosuppressive treatment (negative predictive value >80%) (87, 107). On the other hand, dogs with SRE/IRE and a fecal S100A12 concentration < 2,700 ng/g are very likely to at least partially respond (i.e., show clinical improvement or remission) to treatment (negative predictive value ~100%) (87, 107). Increased fecal S100A12 levels were also linked to individual patient outcomes in canine SRE/IRE (87, 188). Careful patient selection for using this marker is also important, given that fecal S100A12 levels can increase with acute gastrointestinal inflammation (183). Fecal S100A12 levels may be more specific for inflammation than fecal calprotectin concentrations, but measurement of fecal S100A12 in dogs is currently not routinely available.

*N-methylhistamine (NMH)* can be measured *in serum, urine, and fecal samples* (189, 190) and, as a stable product of histamine metabolism, reflects mast cell activation and degranulation in canine CIE (190–192). Increased urine NMH concentrations are detected in ~40% of dogs with CIE and correlate with the severity of histologic changes, but not the numbers of mast cells in the duodenum or the clinical disease severity (189, 190). Further studies are needed to determine the clinical utility of measuring NMH levels in dogs with CIE.

*Serum 3-bromotyrosine* (3-BrY), a stable metabolite of eosinophil peroxidase, is a biomarker of eosinophilic inflammation (193). Dogs with CIE have increased serum 3-BrY concentrations, likely reflecting an eosinophilic component or mixed inflammatory infiltrate (194). Requiring anti-inflammatory or immunosuppressive treatment (i.e., SRE/IRE diagnosis) was associated with higher serum 3-BrY concentrations than responding to an elimination diet (i.e., FRE) (195), but the diagnostic accuracy of serum 3-BrY and corresponding cut-off levels to differentiate CIE subgroups remain to be determined.

*Serum soluble RAGE* (sRAGE) as an anti-inflammatory decoy receptor can sequester ligands such as DAMP molecules (e.g., S100A12) and abrogate cellular proinflammatory RAGE responses (196, 197). Dogs with CIE have an upregulated intestinal transmembrane RAGE expression (198) and decreased serum sRAGE levels that correlate weakly with histologic lesions in the duodenum but not with the severity of clinical signs or patient outcomes (188, 197). With serum sRAGE levels increasing (i.e., normalizing) only in CIE dogs that achieve complete clinical remission, serum sRAGE may be a useful surrogate marker to determine the response to treatment (188). However, further research is needed to confirm the clinical utility of serum sRAGE as a biomarker in canine CIE and determine if sRAGE/RAGE pathways present a potential novel therapeutic target in dogs with chronic gastrointestinal inflammation (198, 199).

## Update/perspective on therapeutic strategies

For some CIE subgroups, research over the last decade led to a change in the role of diet and recommended dietary strategies. Demonstrating the existence of food-responsive PLE (FR-PLE) and several effects of diet on the intestinal microbiome and metabolome (e.g., influence on fecal concentrations of secondary bile acids and abundances of *Peptaceobacter* [*Clostridium*] *hiranonis*) emphasizes the importance of selecting an optimal dietary strategy for dogs with CIE.

### Strategies for PLE

**Figure 5:**
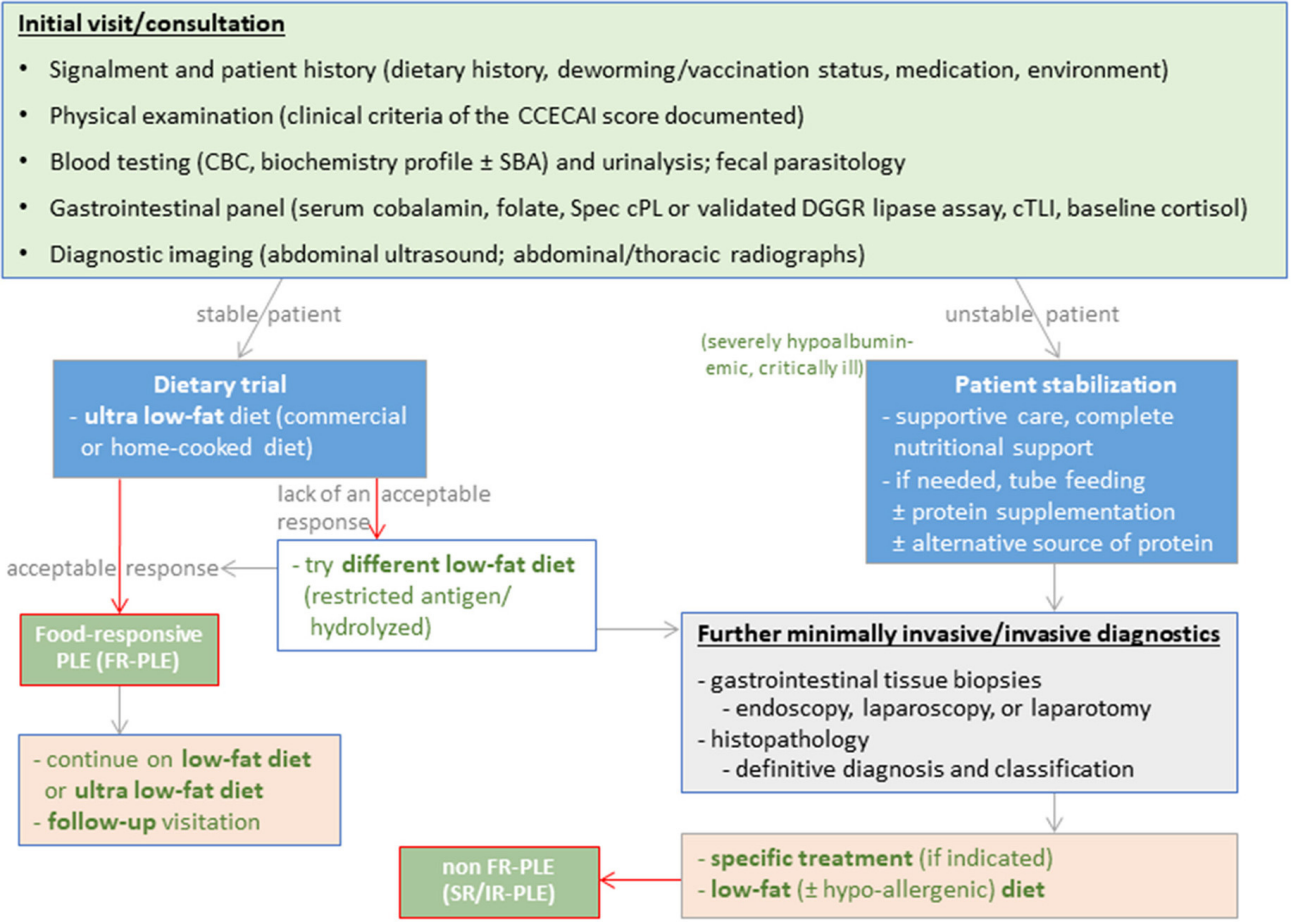
Diagnostic and therapeutic approach to dogs with protein-losing enteropathy (PLE).

Disease severity and chronicity dictate the diagnostic and therapeutic approach to dogs suspected of having protein-losing enteropathy (PLE). It requires sequential laboratory tests, diagnostic imaging, and often more invasive diagnostics (Figure 5). Increased fecal α_1_PI concentrations can detect PLE even before affected dogs develop clinical or clinicopathologic signs (106). Hypoalbuminemia is of prognostic relevance in dogs with PLE (25, 158), and hypovitaminosis D is common (200).

The cornerstone of PLE treatment is lifelong dietary management with a high-protein, low-fat diet ( ≤ 20% fat on a caloric basis or ≤ 20 g fat per 1,000 kcal) (201–203), which can break the cycle of intestinal protein leakage and catabolism. No clinical, clinicopathologic, histologic, or other markers can currently predict food-responsiveness in dogs with PLE (FR-PLE) (40). Serum CRP and fecal calprotectin levels often increase in dogs with PLE as with CIE, particularly those requiring immunomodulatory treatment (108), but both markers add little value to current diagnostic algorithms for PLE.

Response rates with the dietary management of non-infectious, non-neoplastic PLE are high (73–85%) (201–203), and the success of this treatment can be even further improved if multiple small meals are given throughout the day (40). Restoration of a positive protein balance is often achieved or supported with an easily digestible ultra-low fat ( ≤ 10 g fat per 1,000 kcal) commercial diet or a home-made ration (e.g., tilapia and rice or low-fat chicken breast, rice, cottage cheese, and white or sweet potato) (40, 202). Patient preferences and appetite will dictate dietary choices. A board-certified nutritionist should be consulted to verify or design a balanced recipe if the owners elect long-term feeding of a home-made diet. Critically ill dogs may require supplemental or exclusive feeding of either a full elemental diet (e.g., Vivonex^1^), semi-elemental diet, or polymeric formula (e.g., Ensure^2^) and the enteral route of feeding can be ensured by a feeding tube (204). Elemental diets can also be given intermittently or, if needed, continuously to dogs with PLE, but this choice can be expensive.

Because there is no clear evidence for an immune-mediated etiology of PLE (40), immunosuppressive treatment should be very carefully considered. Dogs with PLE that show a marked inflammatory infiltrate on intestinal tissue biopsies and lack an adequate clinical response to dietary intervention within 2–4 weeks should be started on anti-inflammatory or immunosuppressive treatment (201, 202). Glucocorticoids (prediso[lo]ne at an initial dose of 1–2 mg/kg PO q12–24h, then attempt to taper by 25% q2–4 weeks) have catabolic, hyperlipidemic, and prothrombotic effects. Thus, other immunosuppressive drugs (e.g., cyclosporine at 5 mg/kg PO q12–24h or chlorambucil at 4–6 mg/m^2^ PO q24h for 1–3 weeks, then taper) might be better first-line steroid-sparing drug choices. Mycophenolate carries the risk of significant GI adverse effects (24) and is generally not a good choice to treat inflammatory PLE (and CIE in general). Combination immunomodulatory treatment is reserved for dogs with inflammatory PLE that are refractory to dietary and mono-drug therapy (23, 205, 206). Supplemental fiber (e.g., psyllium) or pro-/prebiotics may benefit dogs with PLE, but further research needs to evaluate these treatment options. Absent an infectious etiology, antimicrobial treatment plays no role in canine PLE (21, 22). The somatostatin analog octreotide can be a beneficial rescue treatment (2–4 μg/kg SQ q8–12h) in dogs with inflammatory PLE that are refractory to immunomodulatory treatment, and a study further evaluating its utility in canine PLE is currently underway.

Supportive care should be offered as clinically indicated. Thromboelastography can identify dogs with PLE and a hypercoagulable state (207). Given the increased thrombotic risk, thromboprophylaxis (clopidogrel, low-dose acetylsalicylic acid, or enoxaparin) might be considered as part of the treatment plan in dogs with PLE (208) though evidence for a clear benefit is currently lacking. Dogs with a suboptimal serum vitamin B_12_ status (< 400 ng/L) should receive supplemental cobalamin (50 μg/kg SC weekly for ≥6 weeks or 50 μg/kg PO q24h for ≥12 weeks) (100). Moderate to marked hypofolatemia might require folic acid supplementation (200–400 μg PO q24h for 7–28 days). Vitamin D supplementation may be considered in patients with marked hypovitaminosis D given its association with negative outcomes (200) but further evidence is needed to determine the benefit (and potential risk) of this strategy. Hypocalcemia and/or hypomagnesemia that cause clinical signs should be corrected (calcium gluconate, magnesium sulfate or magnesium chloride). The benefits and risks of supporting plasma oncotic pressure (i.e., synthetic colloids, canine plasma, human or canine albumin) should be carefully weighed in dogs with severe hypoalbuminemia and marked cavitary effusion.

### Probiotics and synbiotics

As microbial imbalances are associated with intestinal inflammation in dogs with CIE, treatments attempting to reduce mucosal inflammation by normalizing dysbiotic states are a rapidly growing research area (Table 3). Probiotics and synbiotics (combinations of a prebiotic and probiotic) are therapies that modify the intestinal microbiota and exert effects on the host immune response to enhance mucosal health. Probiotics are defined as live microorganisms that, when consumed in adequate quantities, confer health benefits on the host (209). Their proposed mechanisms of action include enhancement of mucosal innate and adaptive immune responses (210, 211), production of antimicrobial substances including defensins (210, 212) and organic acids (213), displacement of intestinal pathogens (214), inactivation of bacterial toxins (215), and/or up-regulation of non-specific cellular defenses including heat shock proteins or the inflammasome (216). Very few of these probiotic effects have been demonstrated in companion animals.

**Table 3:** General treatment approaches to intestinal dysbiosis in dogs with CIE [data from (14)].

**Treatment approach**	**Comments**
Dietary trial—hydrolyzed diet or select protein diet	Well tolerated by most dogs; trials with more than one diet may be necessary; avoid dietary sensitivity
Prebiotics/fibers—psyllium, MOS or FOS, pectin	Converted to SCFAs and promote the growth of beneficial bacteria; normalize motility; bind luminal BAs and other irritants
Probiotics—live microbes that are beneficial to host health; prebiotic + probiotic is a synbiotic product	Have strain-specific activity; what strain(s) work best? Side effects are rare; expensive to use
Antimicrobials—may reduce total bacterial loads; may eradicate mucosal bacteria	Cause acute and long-term changes in intestinal microbiota; concern for AMR
Fecal microbiota transplant—provides a functional fecal microbiota from a healthy donor for transfer to a recipient	Donor selection? Optimal means of FMT delivery? Complex dysbiosis of chronic inflammation is difficult to treat and may recur

MOS, mannan oligosaccharides; FOS, fructooligosaccharides; AMR, antimicrobial resistance; FMT, fecal microbiota transplant; BAs, bile acids; SCFAs, short chain fatty acids.

Peer-reviewed publications investigating the use of probiotics or synbiotics in dogs with CIE are sparse (Table 4). The potential benefits of these supplements have been investigated using both clinical trials and *ex vivo* experiments. Clinical studies have included three randomized controlled trials for dogs with FRE (29, 33, 218), two trials for dogs with idiopathic IBD (28, 220), and one trial for dogs with tylosin-responsive diarrhea (ARE) (122). Additionally, two trials have involved *ex vivo* stimulation of endoscopic duodenal biopsies from dogs with FRE (219) or CIE (217). Interpretation of the results and comparison between studies is difficult for varied reasons: (1) probiotic effects are strain-specific, and the documented effects will depend on the specific strain used, which was not always specified, (2) different probiotics were used in different studies to treat different forms of CIE, (3) composition of the vehicle used for a specific probiotic could influence probiotic effects, and (4) most studies were substantially underpowered (221, 222).

**Table 4:** Summary of published studies investigating probiotic or synbiotic use in dogs with CIE.

**Probiotic/synbiotic**	**Disorder**	**Dogs**	**Outcome**	**References**
Probiotic	CIE	12 dogs	Probiotic increased regulatory cytokine expression in *ex vivo* duodenal cultures of dogs with CIE	Sauter et al. (217)
LAB mixture^a^		(compared to 4 healthy dogs)		
Probiotic	ARE	14 dogs with tylosin responsive diarrhea	Relapse of diarrhea after tylosin was discontinued	Westermarck et al. (122)
*L. rhamnosus*				
Probiotic	FRE	21 dogs	Remission observed in both cohorts; no probiotic effect on cytokine expression	Sauter et al. (218)
LAB mixture^a^		(10 on diet; 11 on probiotic)		
Synbiotic *E. faecium* FOS + gum Arabic	FRE	17 dogs (compared to 11 healthy Beagles)	No cytokine protein changes in *ex vivo* duodenal cultures of dogs with FRE; synbiotic increased TNF-α mRNA in whole blood	Schmitz et al. (219)
Probiotic	SRE	20 dogs	Probiotic reduced clinical scores and CD3+ cells; probiotic increased TGF-β cells and TJP (occludin) in duodenal biopsies	Rossi et al. (28)
LAB mixture^b^		(10 on LAB mixture; 10 on metronidazole and prednisolone)		
Synbiotic	FRE	12 dogs	No synbiotic effect on clinical scores, endoscopy, or histology	Schmitz et al. (29)
*E. faecium*		(7 on synbiotic; 5 on diet)		
FOS + gum Arabic				
Probiotic	SRE	26 dogs	No probiotic effect on clinical scores or histology; probiotic increased TJP (multiple) in duodenal and colon biopsies	White et al. (32)
LAB mixture^b^		(14 on probiotic and prednisone; 12 on prednisone and diet)		
Probiotic	SRE	20 dogs	Probiotic (yeast) improved clinical scores and reduced defecation frequency	D' Angelo et al. (220)
*S. boulardii*		(compared to 4 healthy dogs)		
Synbiotic	FRE	12 dogs	No synbiotic effect on fecal microbial diversity	Pilla et al. (33)
*E. faecium*		(7 on synbiotic; 5 on diet)		
FOS + gum Arabic				

CIE, chronic inflammatory enteropathy; ARE, antibiotic-responsive enteropathy; FRE, food-responsive enteropathy; SRE, steroid-responsive enteropathy; FOS, fructooligosaccharides; TJP, tight junction protein.

LAB mixture^a^ contains: *L. acidophilus* (2 strains)*, L. johnsonii*.

LAB mixture^b^ = VSL#3 contains: *L. casei, L. plantarum, L. acidophilus, L. delbrueckii* spp. *bulgaricus, B. longum, B. breve, B. infantis, Scc. salivarus* spp. *Thermophiles*.

In separate clinical trials involving dogs with FRE, use of a synbiotic (*E. faecium*, FOS, and gum Arabic) (29) or a lactic acid bacteria (LAB) mixture of three strains of *Lactobacillus* spp. (218) failed to improve clinical outcome or proinflammatory intestinal cytokine patterns compared to placebo (elimination diet). This same synbiotic did not significantly alter fecal microbiota richness or diversity in dogs with FRE when administered over 6 weeks (33). In dogs with tylosin-responsive diarrhea, the administration of *L. rhamnosus* GG did not extend an absence of GI signs once clinical remission was achieved and the antibiotic was discontinued (122).

In canine idiopathic IBD, probiotic therapy has resulted in modulation of host responses and enhancement of the intestinal epithelial barrier integrity. In one trial, probiotic therapy with VSL#3 (Visbiome, an eight-strain LAB mixture) was investigated in comparison to combination drug therapy (28). Dogs treated with the probiotic showed remission accompanied by changes in tolerogenic mucosal responses (increased numbers of FoxP3^+^ and TGF-β^+^ regulatory T cells) and increased mucosal expression of the tight junction protein occludin. In a separate trial, probiotic mixture VSL#3 and hydrolyzed diet was administered to dogs with idiopathic IBD and compared to a treatment regimen using hydrolyzed diet and prednisone. While both treatment groups saw remission, probiotic therapy was associated with increased beneficial mucosal bacteria and up-regulated expression of several tight junction proteins suggesting improved intestinal barrier function (32). Finally, the yeast probiotic *Saccharomyces boulardii* (as part of a synbiotic preparation) improved clinical severity and stool frequency at days 45 and 60 when administered to IBD dogs with and without PLE (220). In the PLE subgroup, all 3 dogs treated with *S. boulardii* showed improvement in serum albumin vs. 2/3 dogs in the placebo group.

Evidence-based data regarding probiotic use for canine CIE is limited and larger clinical trials are needed. The significant decrease in clinical scores of most dogs with CIE treated with probiotics is best explained by a positive response to dietary intervention. It remains possible that additional health benefits beyond the clinical effects of probiotics might be realized, including systemic immune modulation (223) and potential inhibition of parasite shedding (224).

### Fecal microbiota transplantation

The efforts to promote recovery of dysbiosis and remission in dogs with chronic GI disease by administration of FMT have been investigated only to a limited extent. Guidelines regarding optimal donor screening and clinical indications, as well as the method of delivery (oral vs. rectal catheter vs. endoscopy) and frequency of administration of FMT, are currently lacking (22). Studies defining the potential role for FMT in dogs with CIE, including SRE/IRE or idiopathic IBD, involve two reports (including a total of 17 dogs) where the severity of clinical disease improved following FMT in most dogs (225, 226). The limitations in these studies were significant and include small sample size, lack of a standardized method of fecal delivery, failure to include a control group, and the lack of rigorous molecular testing to assess fecal microbial populations.

## Emerging concepts and future directions

New concepts that have emerged from recent studies on the microbiome-host-interaction, metabolic pathways, and crosstalk within the mucosal immune system are expected to pave the way to a large spectrum of novel treatment strategies that are tailored to the individual patient. Digital health options and artificial intelligence algorithms will likely also play a role in the management of dogs with CIE. Novel diagnostics have been developed, but the clinical utility of these tests and their utility, when included in a panel of tests, remains to be critically evaluated in the future. A new direction is also the further evaluation of gastrointestinal endocrine pathways and the gut-brain axis in canine CIE.

### 2D and 3D organoids for regenerative medicine and drug discovery

Accumulating evidence in humans suggests that intestinal epithelial cell (IEC) dysfunction plays a crucial role in the pathogenesis and progression of IBD (227, 228). Different studies have emphasized the role of MH with the regeneration of IECs in IBD remission (228) and that alterations in gene expression and DNA methylation might persist in IECs despite endoscopic and histologic remission, which might influence the course of disease (229, 230). The absence of drugs that directly influence IEC function and the use of immortalized cell lines or animal models that have limited translatability to human IBD have fostered the development of primary IEC cultures called intestinal organoids: enteroids (small intestine) and colonoids (colon). These long-term *ex vivo* human 3D enteroid/colonoid structures, derived from human adult intestinal stem cells (ISCs), have tremendous potential to recapitulate *in vivo* physiologic conditions used for fundamental research, precision medicine, regenerative medicine, and drug discovery. Under appropriate culture conditions, enteroids/colonoids produce all cell types normally found within the intestinal epithelium. Dogs represent a unique large animal model that naturally develops CIE, sharing remarkable similarities in etiology, clinical course, histologic lesions, and interventional strategies to human IBD (231). The advantages of the dog in relation to other common animal models of intestinal inflammation include their large body size, longer life span, spontaneous (natural) development of CIE, and they possess a GI tract of comparable size, structure, and function to that of humans.

Canine intestinal organoids derived from healthy dogs and dogs with chronic GI diseases have been recently investigated (232–236). In a seminal study, derivation of canine intestinal organoids was described using tissues obtained endoscopically from the duodenum, ileum, and colon of healthy adult dogs and dogs with idiopathic IBD or intestinal mast cell tumor (232). Using an optimized culture protocol, organoids were extensively investigated for their phenotypic characteristics using H&E histology, immunohistochemistry (IHC), RNA *in situ* hybridization (RNA-ISH), and transmission electron microscopy (TEM) for comparison to whole jejunal tissue. Moreover, functional assays evaluating cellular metabolic changes (i.e., optical metabolic imaging [OMI]), transmembrane chloride ion conductance *via* activation of chloride channels in intestinal epithelial cells (cystic fibrosis transmembrane conductance [CFTR] assay), and uptake and transport of parasitic exosomes in response to exposure to the swine nematode, *Ascaris suum*, were performed. There were no differences in morphology of organoids between healthy dogs and dogs with idiopathic IBD visualized by bright-field microscopy. Ultrastructurally, TEM identified features of differentiated cell types, microvilli, and intercellular structures (i.e., adherens junctions, tight junctions, and desmosomes) between epithelial cells in both enteroids and native jejunum. Characterization of molecular markers and IEC subtypes in enteroids and tissues using RNA-ISH/IHC showed that canine enteroids and intestinal tissue share similar phenotypic expression and cell types (Table 5). Lastly, OMI distinguished and quantitated the cellular metabolism between young, rapidly growing enteroids and aged enteroids. Forskolin-induced swelling of enteroids was observed, indicating functional CFTR chloride channels, like in human colonoids (233). Finally, parasitic exosomes were readily absorbed by enteroids demonstrating their successful passage across the epithelium into the organoid lumen as a measure of absorptive capacity and barrier integrity. The same authors also reported development of a polarized 2D epithelial interface (monolayer) of canine biopsy-derived intestinal organoids to study epithelial-luminal interactions (234, 235). This model system confirmed the polarized expression of the P-glycoprotein efflux pump, offers a standardized system for measuring epithelial barrier function, and allows access to the apical side of the epithelium for investigating drug absorption and metabolism, toxicity testing, and host-microbe crosstalk. Another study has investigated the growth and differentiation of intestinal organoids derived from the canine duodenum, jejunum, and colon (236). Different culture conditions optimized organoid growth in a refined (vs. differentiation) medium whereby long-term organoid expansion and differentiation could be maintained. Future organoid technologies (organoid 2D monolayers in transwell) are currently being modeled and will allow for development of new therapeutic options (drug discovery) and improved understanding of gastrointestinal diseases in dogs and other companion animal species.

**Table 5:** Phenotypic/functional characterization of canine organoids and whole jejunum [modified from (237)].

**Characterization**	**Tissue/enteroid**	**Location**
**Marker/cell type**		
Keratin/epithelium	Jejunum, enteroid	Epithelium
PAS/goblet cell	Jejunum, enteroid	Epithelium
Chromogranin A/enteroendocrine cell	Jejunum, enteroid	Epithelium
Vimentin/mesenchymal cell	Jejunum	Submucosa, lamina propria, muscularis
Actin/mesenchymal cell	Jejunum	Lamina propria, muscularis
c-Kit/leukocyte	Jejunum	Resident leukocytes
CD3/T lymphocyte	Jejunum	Lamina propria, intraepithelial lymphocytes
Lysozyme/Paneth cell	No expression	Epithelium
**Functional assays**		
Optical metabolic imaging (OMI)	Enteroids	Epithelium
Cystic fibrosis transmembrane conductance (CFTR)	Enteroids	Epithelium
Parasitic exosome uptake	Enteroids	Epithelium

PAS, periodic acid Schiff.

### Digital health

Raising the bar on the standard of care in canine CIE will involve introducing novel digital technologies. *Artificial intelligence* (AI) describes a variety of digital applications where human tasks (e.g., decision trees) are performed computer-based (238). As a particular AI application, *machine learning* (ML) algorithms can be developed to predict data (e.g., disease, disease subclassification) or outcomes based on previously generated patient data (239). The availability of novel biomarkers in addition to established clinical, endoscopic, and histologic scoring systems lends itself to integrating all these variables (potentially together with the results of other diagnostics such as abdominal ultrasonography) into meaningful ML algorithms (240) for the diagnosis and subclassification of canine CIE or the prediction of individual outcomes. It also offers the potential to establish an ML algorithm for determining the optimal treatment plan and predicting the individual response to treatment and patient outcome (241). Tailoring the diagnostic work-up, accurate ML algorithms may also reduce the overall veterinary healthcare costs for the diagnostic evaluation of the patient and promote an evidence-based medicine approach.

*Digital health* refers to a conceptional infrastructure that combines different digital technologies (242). The use of digital health structures in human medicine has been shown to promote positive health outcomes *via* accessible and efficient delivery of healthcare services and remote patient monitoring (242). Because digital health is particularly useful for monitoring and supporting chronically diseased patients (e.g., through mobile-based remote monitoring applications *via* smartphone), dogs with CIE and their pet owners are expected to benefit tremendously from such services. The enormous potential of these tools (like AI) is likely to unfold in supporting pet owners throughout disease management for improved patient monitoring, owner compliance, and patient outcomes (242). Patient-side biomarker assays (e.g., at-home fecal inflammatory biomarker monitoring), telemedicine and telemanagement (e.g., mentoring) services, and monitoring options (e.g., *via* a web-based diary function) would be very valuable additional tools in canine CIE management. An online survey at the University of Leipzig, Germany that included a cross-sectional questionnaire *Disease tracker app for the management of chronic gastrointestinal disease in dogs and cats—a pet owner's perspective* and yielded 2,416 completed votes (85% of respondents were GenZ/GenY < 35 years old, 78% females) revealed those aims to be aligned with most pet owners' expectations. Top-ranking net promoter scores (NPS) in this questionnaire were obtained for the possibility to be reminded of recurring treatments at home (e.g., weekly cobalamin injection), exchange of medical data with the veterinarian, and receiving individualized treatment recommendations and monitoring instructions. The motivation for most pet owners to use this digital health app was a simple and intuitive design, effective integration of this tool into the daily routine, and improved health management of their pet.

### Individualized medicine

#### Diagnostic panels

Several biomarkers evaluated over the last two decades appear clinically useful surrogate tools for guiding the management of dogs with CIE. Some functional biomarkers (e.g., serum cobalamin or fecal α_1_PI concentrations) are clinically routinely used in dogs with suspected CIE. Genomic biomarkers are currently not readily available but hold great promise for an individualized approach to risk assessment, treatment, and monitoring in canine CIE. Of the inflammatory biomarkers, only serum CRP is currently widely offered, but a human fecal calprotectin assay holds promise for use with specimens from dogs (182, 184), increasing the diagnostic armamentarium in canine gastroenterology.

For the next decade, clinical biomarker research in canine CIE should focus on prospective longitudinal studies to critically evaluate the utility of currently established biomarker assays in dogs with CIE in the routine clinical setting. Incorporating the information gained from several markers into algorithms of “individualized biomarker panels” [as reported for the combination of serum CRP and fecal calprotectin concentration (108)] is expected to further increase the diagnostic accuracy of each individual biomarker and eventually culminate in a holistic individual approach to affected dogs *via* ‘liquid biopsy' (243).

#### Therapeutic strategies

Presenting a multifactorial disease with complex pathogenesis renders canine CIE inherently difficult for determining the individual risk of disease development, optimal treatment strategy, and predicted course of the disease. Beyond extending the diagnostic armamentarium, novel pathway-specific treatment options would provide an improved strategy to manage affected dogs. Three-dimensional (3D) models of canine CIE with complete cytodifferentiation from intestinal stem cells have been developed and are currently being refined allowing to reduce the complexity of the intestinal mucosa to modular functioning microphysiological systems (234, 236). These systems allow for individual or cross-pathway analyses (e.g., to further study the immune components in the pathogenesis of CIE), analysis of the interaction with luminal components (particularly with an “inside-out” enteroid model), exploration and validation of novel therapeutic targets (e.g., alternative anti-inflammatory drugs, certain probiotic strains, and even combinations of different treatment options) (237). Beyond implementing the 3R principle in research, biomimetic gut-on-a-chip technology (e.g., OrganoPlate platform^3^) allows to specifically model aspects of canine CIE that cannot be evaluated *in vivo*, in existing animal models, or in 2D-cell culture systems (e.g., barrier function, complex imbalance in the mucosal immune response) (244) and to apply gene-editing tools (e.g., CRISPR-Cas) (245). These innovative approaches to a “canine-ized” model are not without challenges and limitations, but a window of opportunity for a future approach to treatment that is fine-tuned to the individual patient (“Dog X's gut-on-a-chip”).

### Systemic complications of CIE

Extraintestinal manifestations of IBD in humans include the joints (polyarthritis), skin (erythema nodosum), or eyes (uveitis, episcleritis), but can also involve other organs, such as the liver (cholangitis, cholangiohepatitis), pancreas (pancreatitis), and lung (246–248). In 1 of every 4 patients (25%) the clinical signs associated with the extraintestinal manifestation of the disease can even occur before any gastrointestinal signs develop (248). The occurrence and prevalence of extraintestinal manifestations in canine CIE, as well as their effect on patient management and prognosis, have not been reported and warrant further study.

## Discussion and conclusions

Tremendous research efforts over the last decade resulted in a better understanding of the multifactorial disease complex of canine CIE and the differentiation of breed-specific enteropathies. Milestones reached in researching aspects of the immunopathogenesis, microbiome, metabolome, hypovitaminoses B_12_ and D, and several biomarkers of inflammation are an essential basis for future routes aiming for an individualized medical approach to canine CIE. Refinement of previously established endoscopic and histologic evaluation and scoring criteria has further advanced the standardized assessment and improved the diagnostic algorithms in dogs suspected of CIE. Future investigations in this area need to validate these scoring systems and develop a consensus definition for the assessment of disease remission. More work is also needed to determine the potential benefits of using probiotics, synbiotics, and FMT in dogs with CIE. Subcategorization of food-responsiveness in PLE has changed the approach to affected dogs, and novel dietary strategies and treatment options for canine PLE are to be explored. Future avenues to further evaluate pathogenetic aspects with a focus on drug discovery will benefit from established canine organoid models. AI algorithms are an avenue to improved patient diagnosis and individualized treatment approaches, and digital health tools will likely play a key role in the future of canine CIE management. Important milestones are thus expected in the next decade of canine CIE research.

## Author contributions

AEJ and RMH contributed equally to the manuscript design, writing, generation of figures/tables, editing, and proofing. Both authors contributed to the article and approved the submitted version.

## Conflict of interest

The authors declare that the research was conducted in the absence of any commercial or financial relationships that could be construed as a potential conflict of interest.

## Publisher's note

All claims expressed in this article are solely those of the authors and do not necessarily represent those of their affiliated organizations, or those of the publisher, the editors and the reviewers. Any product that may be evaluated in this article, or claim that may be made by its manufacturer, is not guaranteed or endorsed by the publisher.

## Footnotes

^1^Vivonex^®^ T.E.N, Nestlé Health Science.

^2^Ensure^®^, Abbott Laboratories.

^3^OrganoPlate^®^ platform designed in a 384-well plate format, Mimetas, Leiden, The Netherlands.
